# Evaluation of Gas-to-Liquid Transfer with Ceramic Membrane Sparger for H_2_ and CO_2_ Fermentation

**DOI:** 10.3390/membranes12121220

**Published:** 2022-12-02

**Authors:** Laure Deschamps, Julien Lemaire, Nabila Imatoukene, Michel Lopez, Marc-André Theoleyre

**Affiliations:** 1 URD Agro-Biotechnologies Industrielles (ABI), CEBB, AgroParisTech, 51110 Pomacle, France; 2 Université Paris-Saclay, CentraleSupélec, Laboratoire de Génie des Procédés et Matériaux, SFR Condorcet FR CNRS 3417, Centre Européen de Biotechnologie et de Bioéconomie (CEBB), 3 rue des Rouges Terres, 51110 Pomacle, France; 3 TMA Process, 51100 Reims, France

**Keywords:** biogas production, hydrogen fermentation, gas-to-liquid transfer, membrane sparger, microbubbles

## Abstract

Hydrogen and carbon dioxide fermentation to methane, called bio-methanation, is a promising way to provide renewable and easy-to-store energy. The main challenge of bio-methanation is the low gas-to-liquid transfer of hydrogen. Gas injection through a porous membrane can be used to obtain microbubbles and high gas-to-liquid transfer. However, the understanding of bubble formation using a membrane in the fermentation broth is still missing. This study focused on the impact of liquid pressure and flow rate in the membrane, gas flow rate, membrane hydrophobicity, surface, and pore size on the overall gas-to-liquid mass transfer coefficient (K_L_a) for hydrogen with gas injection through a porous membrane in real fermentation conditions. It has been shown that K_L_a increased by 13% with an increase in liquid pressure from 0.5 bar to 1.5 bar. The use of a hydrophilic membrane increased the K_L_a by 17% compared to the hydrophobic membrane. The membrane with a pore size of 0.1 µm produced a higher K_L_a value compared to 50 and 300 kDa. The liquid crossflow velocity did not impact the K_L_a in the studied range.

## 1. Introduction

The need for renewable low-carbon energy is increasing with global warming. The fast growth of renewable electricity production in Europe, such as solar and wind energy, which are variable and uncontrollable, requires an increase in storage capacity to meet the variable electricity demand. However, electricity is relatively hard to store, which is why power-to-gas technologies are suggested for electricity conversion into renewable gases, which seem to be a promising low-carbon energy carrier with a large storage capacity. First, it is possible to produce hydrogen via water electrolysis with surplus electricity from renewable energy [1]. Second, hydrogen can be further converted with carbon dioxide into biomethane, which can be stored and distributed in the natural gas grid. This reaction can be catalyzed by methanogen archaea according to the reaction CO_2_ + 4 H_2_ → CH_4_ + 2 H_2_O [2], which converts 80% of the hydrogen energy content into methane (based on a higher heating value) and the remaining 20% is released by the exothermic reaction.

This biomethane production requires the dissolution of gases for microorganisms’ assimilation. However, hydrogen has low solubility in water (17 × 10^−6^ Nm^3^·m^−3^·bar^−1^ in water at 25 °C compared to 540 × 10^−6^ Nm^3^·m^−3^·bar^−1^ for carbon dioxide [3]). The low gas-to-liquid transfer of hydrogen is the main limitation of the development of bio-methanation bioprocesses [4]. Moreover, hydrogen consumption yield must be high to limit hydrogen residue in biogas.

Three technologies are currently being studied to increase the gas-to-liquid transfer in the bio-methanation reactor: Continuous stirred-tank reactors, trickle-bed reactors, and membrane reactors. In this study, we focus on the membrane reactor, which is a promising technology for high gas-to-liquid transfer and low energy demand [5,6]. Two strategies are used with the membrane reactor for hydrogen transfer. The first one uses biofilms formed on the membrane surface, which directly consume hydrogen injected into the membrane lumen [7,8]. It results in a 100% hydrogen consumption yield but requires a large surface area due to the progressive gas-to-liquid transfer decrease because of the biofilm [7]. The second uses a membrane to produce microbubbles with possible biogas recycling [5,9]. It results in a high K_L_a value but can lead to residual hydrogen in upgraded biogas [9]. In this work, an external microporous membrane for hydrogen and carbon dioxide transfer was investigated. An anaerobic digestion medium was recirculated into the tubular porous membrane channels while hydrogen and carbon dioxide were injected into the shell side. In previous work, this technology has been shown to lead to a hydrogen transfer yield higher than 99%, which produces biogas with more than 97% methane content [10]. Moreover, previous studies on bubble formation in water with a porous membrane showed that it was a promising way to generate microbubbles to increase the gas-to-liquid transfer [11,12,13,14]. 

In the multiple studies using membranes for hydrogen injection in a bioreactor, gas-to-liquid transfer remains the limiting factor [5,6,9,10]. It is not possible to increase the gas-to-liquid transfer by only increasing the hydrogen flow rate since it will result in higher residual hydrogen in the biogas. Moreover, increasing the gas-to-liquid transfer by increasing the stirring is also limited since the energy demand of the process must remain lower than the energy produced by the process. Thus, it is important to gain knowledge on the impact of parameters, which could improve the gas-to-liquid transfer while limiting the energy demand of the process.

It has been shown that the bubble size was lower when reducing the membrane pore size [12], membrane and medium contact angle [13], transmembrane pressure (TMP) [12,14], and surface tension [13] and increasing the shear stress [13]. However, these parameters had been studied in water. It is well known that the gas-to-liquid transfer is highly affected by the medium composition.

This study aimed at investigating the impact of different parameters on the overall gas-to-liquid mass transfer coefficient (K_L_a) with gas injection through a porous membrane in real fermentation conditions in order to optimize the energy requirement of the methanation process. The parameters studied were the liquid pressure and flow rate in the membrane, gas flow rate, membrane hydrophobicity, and surface and pore size.

## 2. Materials and Methods

### 2.1. Pilot Plant Description

The membrane bioreactor setup was described previously [10]. The total reactor volume was 170 L with a working volume of 150 L and an internal diameter of 0.4 m. The medium height was 1.2 m. The bioreactor was fed continuously with industrial wastewater and operated as an anaerobic membrane bioreactor [10]. Anaerobic digestion of the wastewater produced approximately one volume of carbon dioxide for 3three volumes of exogenous carbon dioxide added for the biomethanation reaction. The internal membrane module used for filtration was composed of hollow fibers made of PVDF (KOCH Separation Solution—France). The membrane surface was 0.5 m² with a pore size of 0.03 µm. Gas recirculation in the filtration membrane module was operated at 15 L·min^−1^ to shake the membrane fibers and prevent membrane fouling. A pump for permeate backflush was used to prevent membrane fouling as well. No sludge was discarded during the experiment except for sampling.

The bioreactor was connected via a liquid recirculation pump to an external membrane module, which can contain one or two ceramic membranes in series. The membranes used were tubular ceramic membranes with 19 channels (Orelis Environnement—KLEANSEP™ BW—Germany, Francfort). Their surface area was 0.25 m². Membranes with different pore sizes were used: 0.1 µm, 300 kDa, and 50 kDa. One 0.1 µm membrane was hydrophobic (fluorinated silane coating by manufacturer). The supplier datasheet is provided in Figure S1.

These membranes were used to dissolve hydrogen and exogenous carbon dioxide in the bioreactor medium. Hydrogen was produced continuously by a water electrolysis device (Peak scientific 450cc—United Kingdom, Inchinnan) and the hydrogen feed flow rate was regulated by a hydrogen flowmeter (Brooks SLA5850—USA, Hatfield, PA). The liquid pressure inside the membrane modules can be regulated with a valve located at their liquid output. Membrane gas and liquid pressures were measured with a pressure gauge.

Bioreactor mixing was ensured by a liquid recirculation at 600 L·h^−1^. A pH and temperature probe (JUMO 201020) and a sludge sample point were placed in the recirculation loop. The temperature was set at 37 °C with a water bath and coil in the bioreactor. The pH was not adjusted in the bioreactor.

### 2.2. Experimental Setup

#### 2.2.1. Preliminary Tests with Water

Preliminary experiments were carried out in water, without microorganisms and thus without hydrogen consumption in order to determine the gas-to-liquid mass transfer coefficient of the membrane. A hydrophobic membrane of 0.1 µm was used for these preliminary tests. The membrane module was fed with tap water to ensure the absence of dissolved hydrogen in the liquid input. The output liquid was discarded. The liquid flow rate was fixed at 1 m^3^·h^−1^ and the liquid pressure in the membrane was atmospheric pressure. Hydrogen was injected at different fixed pressures (between 0.4 and 1.0 bar_g_) controlled by the water electrolysis device. It was ensured that no gas bubbling occurred in the membrane. The dissolved hydrogen flow rate was measured with the hydrogen flowmeter.

#### 2.2.2. Gas-to-Liquid Mass Transfer Coefficient Estimation with Microorganisms

The aim of this study was to determine the impact of the liquid pressure, crossflow velocity, gas feed flow rate, membrane hydrophobicity, and surface and pore size on gas-to-liquid transfer. The evaluation of the gas-to-liquid mass transfer coefficient was performed by carrying out experiments with microorganisms. 

The bioreactor was operated in anaerobic digestion coupled with in situ bio-methanation for several months before this experiment [10]. Then the bioreactor was fed with wastewater at a flow rate of 25 L·day^−1^ corresponding to a hydraulic retention time of 6 days (~1.6 kg_COD_·m^−3^·day^−1^). The feed flow rate of the gaseous phase was fixed at 15 NL·h^−1^ for hydrogen and 3 NL·h^−1^ for carbon dioxide. One liquid gauge was placed on the liquid side of the membrane and one on the gas side to measure the TMP.

Approximately 1 NL·h^−1^ of carbon dioxide was produced from wastewater digestion. The fermentation broth flow rate in the membrane was 0.2 m^3^·h^−1^, which corresponded to a crossflow velocity of 0.30 m·s^−1^, and the liquid pressure was 1.5 bar_g_. For each condition, K_L_a was calculated using Equation (9). While the low gas-to-liquid transfer of hydrogen is the main limitation in the development of bio-methanation bioprocesses [4], only the K_L_a of hydrogen was studied.

In the beginning, a hydrophobic membrane of 0.1 µm was used for the hydrogen injection, and a hydrophilic membrane of 0.1 µm was used for the carbon dioxide injection. The impacts of liquid pressure and crossflow velocity were evaluated by ranging the pressure from 0.5 bar_g_ to 1.5 bar_g_ and ranging the crossflow velocity from 0.30 m·h^−1^ to 0.53 m·h^−1^. Then the impact of the hydrogen feed flow rate was investigated by ranging it from 11 NL·h^−1^ to 15 NL·h^−1^.

The effects of the hydrogen partial pressure, membrane hydrophobicity, and surface and pores size were studied several months later with the same inoculum being reactivated. The inoculum was reactivated for 1 month with the constant feeding of carbon dioxide and hydrogen using a hydrophobic membrane for hydrogen injection and a hydrophilic membrane for carbon dioxide injection. Then hydrogen and carbon dioxide were injected together into the membrane module; first in one hydrophobic membrane of 0.1 µm and then in one hydrophilic membrane of 0.1 µm for the evaluation of the membrane hydrophobicity impact on the gas-to-liquid transfer. Then two hydrophilic membranes of 0.1 µm were used in series for the evaluation of the membrane surface effect on the gas-to-liquid transfer. Finally, two membranes of 300 kDa and 50 kDa were used consecutively to investigate the impact of the membrane pore size on the gas-to-liquid transfer.

The parameters applied in each condition are summarized in Table 1.

### 2.3. Analytical Procedure

During all operations, biogas composition was recorded twice a day with microGC. Volatile fatty acids (VFA) were measured 3 times a week by HPLC. The chemical oxygen demand (COD) was measured 3 times a week. Mixed-liquor suspended solids (MLSS) and mixed-liquor volatile suspended solids (MLVSS) analyses were performed once a week. The analytical procedure details for biogas composition, VFA, COD, MLVSS, and MLSS have been described previously [10]. Online data acquisition was carried out for the weight of the produced permeate, the volume of the produced biogas, and the pH value. An online data acquisition system and equipment references have been described previously [10].

### 2.4. Theoretical Calculations

The hydrogen consumption yield ($\eta_{H_{2}}$) was calculated as follows:

(1) $$\begin{array}{c} \eta_{H_{2}}=\frac{{\dot{G}}_{H_{2}-\;\mathrm{IN}\;}-{\dot{G}}_{H_{2}-\;\mathrm{OUT}\;}}{{\dot{G}}_{H_{2}-\;\mathrm{IN}\;}} \end{array}$$

where ${\dot{G}}_{H_{2}-\;\mathrm{IN}}$ is the volumetric hydrogen feed flow rate (Nm^3^·h^−1^) and ${\dot{G}}_{H_{2}-\;\mathrm{OUT}}$ is the volumetric hydrogen effluent rate.

**Figure 1:**
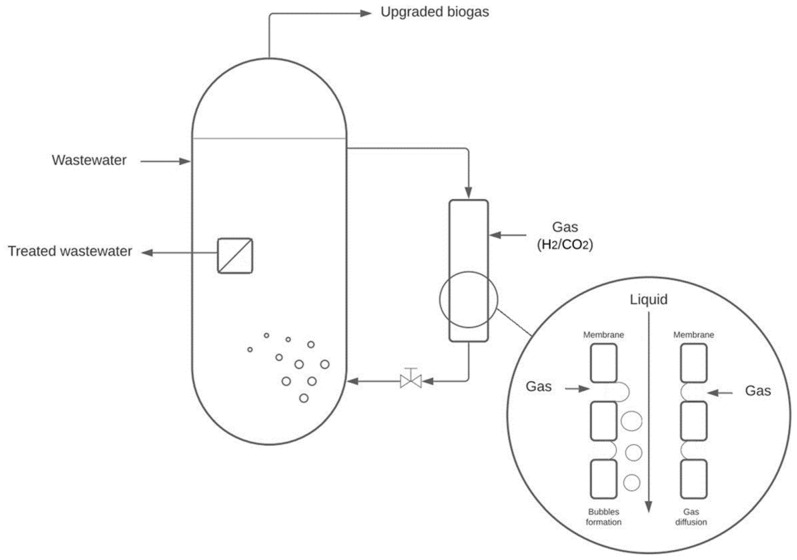
Schematic representation of gas-to-liquid transfer in the membrane bioreactor.

The membrane used for the gas-to-liquid transfer acts as a membrane contactor by dissolving the hydrogen directly in the membrane module and a membrane sparger by forming microbubbles on the membrane surface, which are then dissolved in the bioreactor acting as a bubble column bioreactor. The schematic representation of the overall gas-to-liquid transfer is shown in Figure 1.

The preliminary experiment carried out with water was used to determine the membrane gas-to-liquid mass transfer coefficient. First, a mass balance on hydrogen along the membrane is used for the mass transfer coefficient calculation. Measurements were performed at a steady state. Assuming there was a concentration gradient of hydrogen in the liquid along the membrane, the following formula can be established:

(2) $$\begin{array}{c} K_{L}a\cdot {Ω}_{Liquid}\cdot l=L\cdot \ln\left(\frac{C^{*}}{C^{*}-C\left(l\right)}\right) \end{array}$$

where $K_{L}a$ is the overall gas-to-liquid mass transfer coefficient (h^−1^), ${Ω}_{Liquid}$ is the liquid membrane section (m^2^), l is the membrane length (m), and L is the liquid flow rate in the membrane (m^3^·h^−1^). $C^{*}$ is the concentration of hydrogen in the liquid phase if saturated (Nm^3^·m^−3^) and C(l) is the concentration of hydrogen in the liquid phase at the output of the membrane (Nm^3^·m^−3^).

A mass balance of hydrogen on the overall membrane gives the following formula:

(3) $${\dot{G}}_{H_{2}-{\;\mathrm{IN}}_{Diffusion}}\;=L\cdot \left(C\left(l\right)-C\left(0\right)\right)$$

where C(0) is the concentration of hydrogen in the liquid phase at the input of the membrane and ${\dot{G}}_{H_{2}-{\;\mathrm{IN}}_{Diffusion}}$ is the volumetric hydrogen feed flow rate directly dissolved on the membrane surface (Nm^3^·h^−1^). Experiments were carried out with water containing no hydrogen in the input water. Thus:

(4) $$\begin{array}{c} C\left(l\right)=\frac{{\dot{G}}_{H_{2}-{\;\mathrm{IN}}_{Diffusion}\;}}{L} \end{array}$$

According to Henry’s law:

(5) $$\begin{array}{c} C^{*}=K_{H_{2}}\cdot P_{H_{2}} \end{array}$$

where $K_{H_{2}}$ is the hydrogen Henry’s constant (Nm^3^·m^−3^·bar^−1^), which is 17 × 10^−6^ Nm^3^·m^−3^·bar^−1^ at 25 °C, and $P_{H_{2}}$ is the hydrogen partial pressure (bar). Consequently, it is possible to measure K_L_ by measuring the hydrogen flow rate dissolved for a given liquid flow rate by combining Equations (2), (4) and (5):

(6) $$\begin{array}{c} K_{Lmembrane}=L\cdot ln\frac{K_{H_{2}}\cdot P_{H_{2}}}{K_{H_{2}}\cdot P_{H_{2}}-\frac{{\dot{G}}_{H_{2}-{\;\mathrm{IN}}_{Diffusion}}}{L}}S \end{array}$$

where *S* is the membrane surface (m²), equivalent to $a\cdot {Ω}_{Liquid}\cdot l$, and $K_{Lmembrane}$ is the overall membrane mass transfer coefficient for hydrogen (m·h^−1^) For the bubble fraction, the global mass transfer coefficient is calculated with a formula adapted from Díaz et al., 2015 [5]. Increasing the partial pressure on hydrogen above 10^−5^ atm in the bioreactor would result in VFA accumulation and COD removal efficiency decreasing due to microbial inhibition [15], which were not observed. During the experiment, the VFA concentration was lower than 10 mg·L^−1^. Therefore, it is assumed that dissolved hydrogen was consumed instantly by the microorganisms, which means that the hydrogen concentration in the liquid phase, far from the gas–liquid interface, was close to zero. 

Compared to the formula proposed by Díaz et al., 2015 [5], the flow of hydrogen dissolved directly on the membrane surface is subtracted from the total flow of hydrogen dissolved and consumed by microorganisms to consider only the bubble fraction.

(7) $$K_{Lbubbles}\cdot a_{bubbles}=\frac{{\dot{G}}_{H_{2}-\;\mathrm{IN}\;}-{\dot{G}}_{H_{2}-\;\mathrm{OUT}\;}-{\dot{G}}_{H_{2}-{\;\mathrm{IN}}_{Diffusion}\;}\;}{K_{H_{2}}\cdot P_{H_{2}}\cdot V}$$

where $K_{Lbubbles}$ is the overall bubble mass transfer coefficient for hydrogen (m·h^−1^), $a_{bubbles}$ is the surface-area-to-volume ratio of bubbles (m^2^·m^−3^), and V is the bioreactor volume (m^3^). $K_{Lbubbles}\;\mathrm{and}\;a_{bubbles}$ were never measured separately.

K_L_a was only measured for hydrogen and not for carbon dioxide. Equation (7) shows that it is necessary to determine the gaseous effluent flow rate. However, the carbon dioxide gaseous effluent flow rate is the sum of endogenous carbon dioxide production and exogenous carbon dioxide injection not dissolved. Endogenous carbon dioxide production could be estimated with previous data but will give an approximation of the real K_L_a for carbon dioxide since variation in endogenous carbon dioxide production can occur with the different batches of wastewater used. However, hydrogen is known to be the limiting factor in the bio-methanation process. In this study, we ensured that pH did not increase to ensure that carbon dioxide is properly dissolved. The link between pH and dissolved carbon dioxide has been explained previously [10]. Carbon dioxide dissolution was not a limiting factor during the entire experiment.

## 3. Results and Discussion

### 3.1. Preliminary Tests

**Figure 2:**
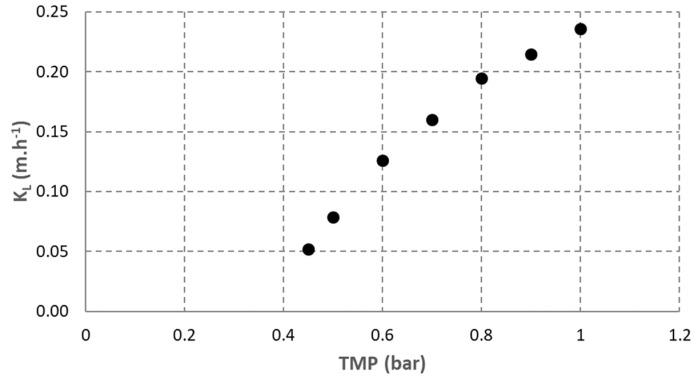
Measurement of membrane mass transfer coefficient at different gas injection pressure.

Results obtained from the preliminary tests carried out with water are shown in Figure 2. The evolution of the membrane gas-to-liquid mass transfer coefficient at different TMP was measured. These results were obtained with gas diffusion only when no bubbles were formed on the membrane surface. At a gas pressure higher than 1 bar_g_, bubbles were observed. The recirculation flow rate was fixed at 1 m^3^·h^−1^ (equivalent to 1.5 m·s^−1^).

The results obtained show that the membrane gas-to-liquid mass transfer coefficient increased with TMP. This is explained by the fact that higher TMP prevents membrane pores from wetting. Indeed, it was reported that in the hollow-fiber membrane contactor, despite membrane hydrophobicity, partial wetting of membrane pores can occur and reduce the mass transfer coefficient for gas diffusion [16]. 

The K_Lmembrane_ measured in these conditions can be used to determine the gas flow rate dissolved on the membrane surface with the following formula obtained from Equation (6):

(8) $$\begin{array}{c} {\dot{G}}_{H_{2}-{\;\mathrm{IN}}_{Diffusion}\;}=\left(1-\frac{1}{e^{\frac{K_{Lmembrane}\cdot S}{L}}}\right)\cdot K_{H_{2}}\cdot L\cdot P_{H_{2}} \end{array}$$

For the rest of the experiments, the liquid pressure was fixed at 1.5 bar_g_, the liquid flow rate was fixed at 0.2 m^3^·h^−1^, and the hydrogen flow rate was fixed at 15 NL·h^−1^. The maximal TMP observed with this membrane was 1 bar during the rest of the experiments, thus K_L_ of 0.25 m·h^−1^ was used for the estimation of the maximal hydrogen flow rate dissolved by diffusion in the membrane. However, *K_Lmembrane_* was likely lower in the rest of the experiments. Indeed, most of the time, TMP was lower than 1 bar. Moreover, a lower liquid velocity was applied compared to preliminary tests. Liquid velocity is known to have a positive effect on K_L_ in membrane contactors. A high liquid velocity reduces the liquid film on the membrane surface, which increases the gas-to-liquid transfer [17]. Moreover, preliminary tests were carried out in water while further experiments were carried out with anaerobic digestion broth. This broth contained microorganisms and other suspended solids, which can also affect negatively K_L_ compared to water [18].

Even with K_L_ of 0.25 m·h^−1^, the maximal dissolved gas flow rate is estimated at approximately 2.1 L·h^−1^, which represents 14% of the hydrogen feed flow rate injected. Then, less than 14% of the injected biogas can be dissolved directly in the membrane contactor. Thus, while the directly dissolved fraction must be low, for the rest of the study, an approximation was performed for the estimation of overall K_L_a by using Equation (7) assuming that the directly dissolved hydrogen is negligible compared to the total assimilated hydrogen:

(9) $$\begin{array}{c} K_{L}a=K_{\mathrm{Lbubbles}}\cdot a_{bubbles}=\frac{{\dot{G}}_{H_{2}-\;\mathrm{IN}\;}-{\dot{G}}_{H_{2}-\;\mathrm{OUT}\;}\;}{K_{H_{2}}\cdot P_{H_{2}}\cdot V} \end{array}$$

### 3.2. Impact of Liquid Pressure

**Figure 3:**
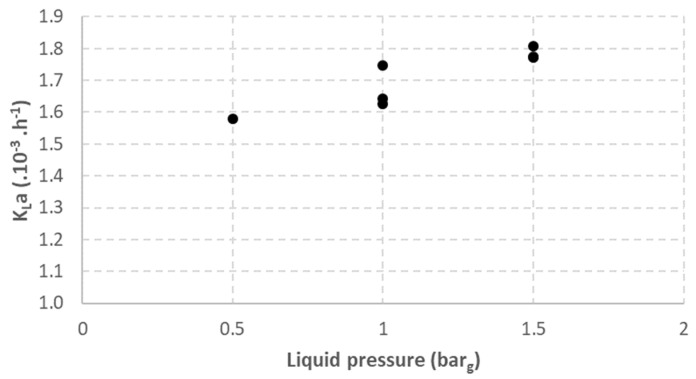
Impact of liquid pressure on K_L_a.

The results obtained from the study regarding the liquid pressure effect on the gas-to-liquid transfer are shown in Figure 3. A positive effect of liquid pressure on K_L_a is observed. K_L_a was only 1.58 × 10^−3^ h^−1^ with a liquid pressure of 0.5 bar and increased to 1.78 × 10^−3^ h^−1^ with a liquid pressure of 1.5 bar. This difference could be explained by the lower volume occupied by hydrogen in the membrane channels with increasing liquid pressure; meanwhile, the gas flow rate was fixed at 15 NL·h^−1^ during the entire experiment. At 1.5 bar_g_, hydrogen occupied approximately 3% of the volume in membrane channels and 5% at 0.5 bar_g_. Indeed, bubble coalescence in the membrane module is less likely at a lower hydrogen volume, leading to smaller bubbles and, finally, better hydrogen assimilation inside the bioreactor.

Moreover, the TMP was 0.8 bar when the membrane liquid pressure was at 1.5 barg while TMP was 1 bar with a liquid pressure of 0.5 barg. Kukizaki and Goto, 2006 [12] showed that bubble production at higher TMP had a higher mean diameter and higher bubble size dispersion (for TMP 2 times higher than the bubbles’ pressure point). This is consistent with the higher hydrogen gas-to-liquid transfer at higher liquid pressure. Indeed, smaller bubbles have a larger surface for a constant volume, therefore increasing the total surface exchange. Moreover, smaller bubbles have lower rising velocity, which increases the gas hold up and increases the total surface exchange [19].

### 3.3. Impact of Liquid Flow Velocity

**Figure 4:**
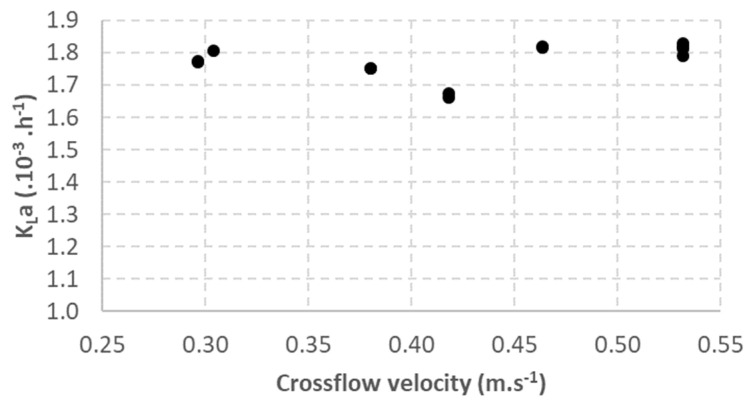
Impact of crossflow velocity on K_L_a.

Crossflow velocity played an important role in bubble formation on the membrane surface. The shear force generated by the liquid crossflow is crucial for bubble detachment from the membrane. The results obtained from the study of the crossflow velocity effect on the gas-to-liquid transfer are shown in Figure 4. The results show that the crossflow velocity had a negligible effect on K_L_a, which remained between 1.66 × 10^−3^ h^−1^ and 1.83 × 10^−3^ h^−1^ over the studied range (0.30 to 0.53 m·s^−1^)_._

Crossflow velocity is required to detach bubbles from the membrane surface. Previous studies showed that bubbles’ mean diameter decreases with increasing velocity until it reaches a minimum bubble diameter. Velocity has almost no effect on K_L_a in the studied range, suggesting that a liquid crossflow velocity of 0.30 m·s^−1^ is sufficient to obtain the minimum bubble diameter, which corresponds to an optimal gas-to-liquid surface exchange with minimal energy consumption in the experimental conditions tested. Indeed, if the minimum bubble diameter was not reached, increasing the crossflow velocity would reduce the bubble diameter, which increases a and then increases K_L_a. 

The minimum droplet diameter is also observed for droplet emulsion formation with the membrane. Two reasons have already been proposed for the minimum droplet diameter reached with high crossflow velocity and can explain the minimum bubble diameter observed. Firstly, the decrease in the bubble diameter at a high crossflow velocity is prevented due to bubbles hindering each other [12,20]. Then, the liquid can flow above the bubbles instead of flowing around the formed bubbles to detach them from the membrane. Secondly, it is also suggested that, with small bubbles, the sublayer thickness is thicker than the bubble diameter, reducing the flow velocity around the bubbles [12,21].

### 3.4. Impact of Gas Flow Rate

**Figure 5:**
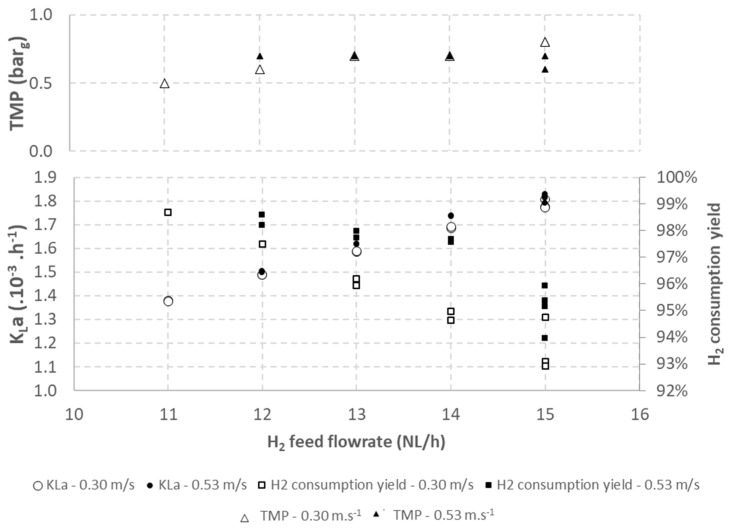
Impact of hydrogen feed flow rate on K_L_a.

The effect of the gas feed flow rate on the gas-to-liquid transfer was studied for two liquid crossflow velocities (0.30 m·s^−1^ and 0.53 m·s^−1^). The results obtained for K_L_a, hydrogen consumption yield, and TMP are shown in Figure 5. 

The results show an increase in K_L_a with a feed flow rate of 0.30 and 0.53 m·s^−1^ for both crossflow velocities. The increase in K_L_a with the feed flow rate was expected while increasing the feed flow rate increases the gas-to-liquid exchange surface (a) by increasing the total volume of gas. However, a decrease in the hydrogen consumption yield was observed with the increase in the gas feed flow rate. This observation is mainly explained by the increasing bubble size. Indeed, if the bubbles’ mean diameter increases, bubbles rise faster in the bioreactor corresponding to lower residence time, so they have a shorter time to be dissolved and consumed in the bioreactor, resulting in higher hydrogen residual content in the headspace.

The increasing mean diameter of bubbles is also consistent with TMP measurements, which increase with an increasing gas feed flow rate. For instance, TMP varied from 0.5 bar for 11 NL_H2_·h^−1^ to 0.8 bar for 15 NL_H2_·h^−1^ for a crossflow velocity of 0.30 m·h^−1^. Indeed, an increase in TMP was already correlated to the formation of bubbles with a higher mean diameter [12].

These results show that increasing the gas feed flow rate to increase K_L_a in a hydrogen or syngas fermentation bioreactor is a limited solution. Indeed, when increasing the gas feed flow rate, another parameter must be optimized to maintain a high hydrogen consumption yield.

### 3.5. Impact of Broth Characteristics

Experiments carried out on membrane hydrophobicity, membrane surface, and membrane pore size were carried out several months after the first experiments. Half of the bioreactor broth was kept and used as the inoculum. Biomass acclimation was performed by working with a hydraulic retention time of 6 days and fed with carbon dioxide and hydrogen for one month. This was performed to ensure that biomass was active, so it did not reduce hydrogen consumption. Then, hydrogen that is not consumed can be attributed only to the gas-to-liquid transfer limit. Stable COD removal efficiency, hydrogen consumption yield, and MLVSS were reached, as well as a low VFA content (<10 mg·L^−1^). 

However, despite the same conditions applied compared to previous experiments (liquid flow velocity of 0.30 m·s^−1^, liquid pressure of 1.5 bar_g_, and gaseous florates of 15 NL_H2_·h^−1^ and 3 NL_CO2_·h^−1^), a lower K_L_a was obtained. K_L_a value was 1.78 × 10^−3^ h^−1^ previously, while it was only 1.45 × 10^−3^ h^−1^ in the second part of the experiment. TMP also changed between these two periods and went from 0.8 bar to lower than 0.1 bar.

The differences observed can be explained by the different characteristics of the bioreactor broth. MLVSS and MLVS were divided into two between two periods. It stabilized at 1.9 g/L during the second part of the experiment while it was at 3.8 g/L in the first part. Higher solids in the first half of the experiment are likely extracellular polymeric substances or soluble microbial particles that generally accumulate in the membrane bioreactor [22]. All of these parameters are known to affect bubble formation. Indeed, they can have a surfactant effect and reduce bubble size. These parameters can also affect the K_L_ but it has been shown that for microbubbles (<1.5 mm), K_L_ is not affected by the surfactant [23]. Therefore, the decrease in K_L_a observed in the second part can be attributed to bigger bubbles forming. Moreover, the higher particle content in the first part might increase the shear stress and promote bubble detachment with a smaller size, which also positively affects K_L_a in the first part compared to the second part.

Furthermore, differences in TMP were observed. These differences may be due to a change in affinity between the membrane and the broth due to surfactants’ accumulation (extra-polymeric substances or soluble microbial particles). A smaller contact angle increases the bubble point pressure corresponding to an increase in the minimal pressure required for bubble formation [24]. The difference in TMP may also be due to partial wetting or fouling of the membrane, which had been used for several months before the first part of the experiment. The membrane was cleaned and dried before the second part of the experiment. Membrane fouling could previously prevent the use of larger pores of the membranes, producing a smaller bubble size and a higher gas-to-liquid transfer. When using a clean membrane, the gaseous phase will preferentially cross the larger pores of the membrane, which will form larger bubbles.

### 3.6. Impact of Hydrogen Partial Pressure

Table 2 shows the impact of hydrogen partial pressure, membrane hydrophobicity, and surface and membrane pore size on the gas-to-liquid transfer. Similar K_L_a values were obtained when hydrogen was injected alone through the 0.25 m² hydrophobic membrane and together with carbon dioxide through the same membrane. However, the difference was observed in terms of the hydrogen consumption rate, which was 76% (equivalent to 11.4 NL_H2_·h^−1^) when hydrogen was consumed alone compared to 63% (equivalent to 9.5 NL_H2_·h^−1^) when it was injected with carbon dioxide. This result means that the difference in the hydrogen consumption flow rate was mainly due to the hydrogen partial pressure reduction in the membrane, which reduced hydrogen partial pressure in the microbubbles formed. Then the driving force of the hydrogen gas-to-liquid transfer was reduced as well.

The decrease in hydrogen consumption can also be explained by the increase in the gaseous injection flow rate, which was 60 L·h^−1^·m^−2^ when hydrogen was injected alone and 72 L·h^−1^·m^−2^ when it was injected with carbon dioxide. Bubbles with larger diameters could be formed when increasing the gaseous flow rate, which could decrease the gas-to-liquid transfer by decreasing the total exchange surface. However, K_L_a was not reduced, which suggests that the total exchange surface is not affected.

This result shows that the injection of hydrogen and carbon dioxide should be carried out in different injection modules in order to maintain a high driving force for hydrogen transfer from bubbles to the liquid phase.

### 3.7. Impact of Membrane Hydrophobicity

The results shown in Table 2 highlight the effect of membrane hydrophobicity on gas-to-liquid transfer. A higher K_L_a of 1.69 × 10^−3^ h^−1^ was obtained with a hydrophilic membrane compared to a hydrophobic membrane where K_L_a was 1.44 × 10^−3^ h^−1^. This resulted in an increase in the hydrogen consumption yield from 63% to 74%. The higher gas-to-liquid transfer obtained with the hydrophilic membrane can be attributed to smaller bubbles forming with this membrane. When the membrane is hydrophilic, its contact angle with the aqueous broth is lower. Thus, it led to smaller bubbles forming because the gaseous phase detached more easily from the membrane pores [13]. Consequently, it is recommended to use a membrane that has a low contact angle with the fermentation broth to optimize the gas-to-liquid transfer by minimizing the bubble size.

However, with the hydrophilic membrane, the TMP was 0.5 bar higher compared to the hydrophobic membrane at the same gas flow rate. This is most likely due to the membrane pores wetting, which led to an increase in the minimum gas pressure required for bubble formation [24]. Thus, the more affinity there is between the membrane and the broth, the higher the TMP will be at a given flow rate. However, a TMP of 0.5 bar is still relatively low compared to the maximal TMP suitable for the ceramic membrane (10 bar). Nevertheless, for some membranes, mainly organic membranes, it would not be possible to apply such TMP without damaging the membrane.

### 3.8. Impact of Membrane Surface

The effect of the membrane surface on the gas-to-liquid transfer can also be observed in the results shown in Table 2. Doubling the membrane surface (0.5 m²) led to a drop in TMP (<0.1 bar instead of 0.5 bar). The lower TMP is fundamentally linked to the lower gas flux (in Nm^3^·h^−1^·m^−2^), which was twice lower when the membrane surface was doubled, as has already been reported [12]. 

Meanwhile, doubling the membrane surface led to an increase in K_L_a from 1.69 × 10^−3^ h^−1^ to 1.89 × 10^−3^ h^−1^, which results in an increase in the hydrogen consumption yield from 74% to 87%. This difference is mainly explained by the smaller bubbles obtained when injecting the same gas flow rate with a larger membrane surface. The lower TMP observed with two membrane modules is consistent with a smaller mean diameter of bubbles and lower dispersion [12], which correspond to a higher total exchange surface and gas hold up in the bioreactor.

### 3.9. Impact of Membrane Pore Size

The effect of the membrane’s pore size on the gas-to-liquid transfer can also be studied from the results shown in Table 2.

First, we observed that K_L_a was lower with a smaller pore size (1.83 × 10^−3^ h^−1^ with a 0.1 µm membrane but approximately 1.60 × 10^−3^ h^−1^ with 300 kDa and 50 kDa membranes). This observation differs from other studies, which showed that a smaller bubble diameter was obtained with a smaller membrane pore size [13,25]. However, these studies were operated at a constant TMP while the gaseous flux was kept constant in the current study. Thus, a lower gaseous flux can be expected with a smaller membrane pore size when TMP is constant due to the increase in membrane resistance, which results in a smaller bubble diameter.

Moreover, previous studies [11,13,14] used membranes with pore sizes ranging from 0.2 µm to 10 µm while the membrane pore size in this study ranged from 50 kDa to 0.1 µm. The gaseous flux, which was between 0.04 and 0.07 m^3^·h^−1^·m^−2^ in our study, is also much lower than in previous studies (between 1 and 40 m^3^·h^−1^·m^−2^). Moreover, bubble coalescence could be a limiting factor to obtaining smaller bubbles with a very small membrane pore size, when many more bubbles are produced and a larger proportion of the membrane porosity is used. 

The different K_L_a observed could also be attributed to lower pore wetting of the membrane when the maximum pore size of the membrane decreases [26]. Membrane wettability has been previously shown to increase K_L_a.

## 4. Conclusions

All experimental results lead to suggestions for the design of an optimal external membrane sparger for hydrogen and carbon dioxide fermentation. A hydrophilic membrane seems more appropriate because its contact angle with the fermentation medium must be low to reduce the bubble size and increase the gas-to-liquid transfer. Then, in the experimental range of hydrogen flux and membrane pore size, a membrane with a 0.1 µm pore size is recommended. Furthermore, the liquid pressure or the membrane surface can be increased to increase the hydrogen consumption yield. Finally, the minimal liquid crossflow velocity, which is related to the minimal diameter of bubbles, must be determined to minimize the energy consumption, depending on the previous operating choice.

## Figures and Tables

**Table 1:** Parameters applied in each condition.

Parameter Studied	Liquid Pressure (bar_g_)	Crossflow Velocity (m·s^−1^)	H_2_ Flow Rate (NL·h^−1^)	CO_2_ Injection	Membrane Hydrophobicity	Membrane Surface (m²)	Membrane Pore Size (µm)	Results Part
Preliminary tests	Atmospheric pressure	1.5	Measured for different gas pressures	None ^a^	Hydrophobic	0.25	0.1	3.1
Liquid pressure	From 0.5 to 1.5	0.30	15	Separated ^a^	Hydrophobic	0.25	0.1	3.2
Crossflow velocity	1.5	From 0.30 to 0.53	15	Separated ^a^	Hydrophobic	0.25	0.1	3.3
Gas flow rate	1.5	0.30–0.53	From 11 to 15	Separated ^a^	Hydrophobic	0.25	0.1	3.4
Broth characteristics	1.5	0.3	15	Separated ^a^	Hydrophobic	0.25	0.1	3.5
Hydrogen partial pressure	1.5	0.3	15	Together with H_2_^b^	Hydrophobic	0.25	0.1	3.6
Membrane hydrophobicity	1.5	0.3	15	Together with H_2_^b^	Hydrophobic and hydrophilic	0.25	0.1	3.7
Membrane surface	1.5	0.3	15	Together with H_2_^b^	Hydrophilic	0.5	0.1	3.8
Membrane pore size	1.5	0.3	15	Together with H_2_^b^	Hydrophilic	0.5	50 kDa, 300 kDa and 0.1 µm	3.9

^a^ Hydrogen represented 100% of volume in the injection membrane. ^b^ Hydrogen represented 80% of the volume in the injection membrane.

**Table 2:** Effect of H_2_ partial pressure, membrane hydrophobicity, and surface and pore size on gas-to-liquid transfer.

Membrane Used	Gas Injected	K_L_a (×10^−3^·h^−1^)	TMP (bar)	H_2_ Consumption Yield (%)
Hydrophobic-0.25 m²-0.1 µm	H_2_ *	1.45 ± 0.01	<0.1	76 ± 1
Hydrophobic-0.25 m²-0.1 µm	H_2_ + CO_2_	1.44 ± 0.09	<0.1	63 ± 4
Hydrophilic-0.25 m²-0.1 µm	H_2_ + CO_2_	1.69 ± 0.01	0.5	74 ± 1
Hydrophilic-0.5 m²-0.1 µm	H_2_ + CO_2_	1.89 ± 0.10	<0.1	83 ± 4
Hydrophilic-0.5 m²-300 kDa	H_2_ + CO_2_	1.61 ± 0.05	<0.1	70 ± 2
Hydrophilic-0.5 m²-50 kDa	H_2_ + CO_2_	1.60 ± 0.03	<0.1	70 ± 1

* Carbon dioxide was injected through another ceramic membrane.

## Data Availability

Not applicable.

## Supplementary Materials

The following supporting information can be downloaded at: https://www.mdpi.com/article/10.3390/membranes12121220/s1, Figure S1: Membrane supplier data sheet; Figure S2: Pilot picture; Table S1: Supplementary data.

## Acronyms

$\eta_{H_{2}}$: Hydrogen consumption yield (%); ${\dot{G}}_{H_{2}-\mathrm{IN}\;}:$ Hydrogen feed flow rate (Nm^3^·h^−1^); ${\dot{G}}_{H_{2}-{\mathrm{IN}}_{Diffusion}\;}:$ Hydrogen flow rate dissolved on the membrane surface (Nm^3^·h^−1^); ${\dot{G}}_{H_{2}-\;\mathrm{OUT}\;}:$ Hydrogen effluent flow rate (Nm^3^·h^−1^); $K_{Lmembrane}$: Overall membrane mass transfer coefficient for hydrogen (m·h^−1^); $K_{Lbubbles}\;:$ Overall bubbles mass transfer coefficient for hydrogen (m·h^−1^); $a_{membrane}$: Surface area to volume ratio of membrane (m^2^·m^−3^); $a_{bubbles}\;:$ Surface area to volume ratio of bubbles (m^2^·m^−3^); K_L_a: Gas-to-liquid mass transfer coefficient (h^−1^)—Equivalent to K_L_.a; ${Ω}_{effective}:$ Membrane section (m^2^); l: Membrane length (m); S: Membrane surface (m^2^); L: Liquid flow rate in the membrane (m^3^·h^−1^); $C^{*}$: Concentration of hydrogen in liquid phase if saturated (Nm^3^·m^−3^); C: Concentration of hydrogen in liquid phase (Nm^3^·m^−3^); $K_{H_{2}}$: Henry’s constant (Nm^3^·m^−3^·bar^−1^); $P_{H_{2}}$: Hydrogen partial pressure (bar); V: Bioreactor volume (m^3^); TMP: Transmembrane pressure (bar).
