# Supplementary material for: Evaluation of Gas-to-Liquid Transfer with Ceramic Membrane Sparger for H2 and CO2 Fermentation
Source: Membranes (Basel). 2022 Dec 2;12(12):1220. doi: 10.3390/membranes12121220 (PMC9783551; doi:10.3390/membranes12121220)
Supplement: Supplementary file 1 [file membranes-12-01220-s001.zip › Figure S1 - Membrane supplier data sheet.pdf]

KLEANSEP™ ceramic membranes and modules

Data sheet

ORELIS environnement
ALSYS Group

Historical ranges market leaders

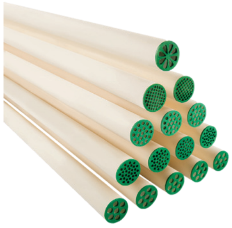
Kleansep™ membranes

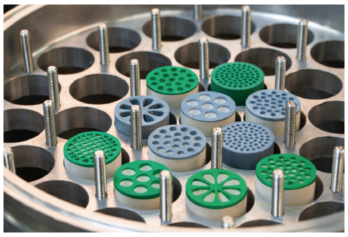
Kleansep™ modules

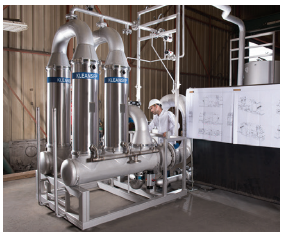
Kleansep™ filtration system

ALSYS ceramic membranes are tube-shaped filters with an asymmetrical membrane structure. With their unique design and construction, KLEANSEP™ products are well-suited to a wide range of applications.

ALSYS experiences in the field of ceramic membranes technology

Recycle Water and Liquids		Process	
Water	Oil & Gas	Chemistry	Feed & Food
Treatment of industrial, domestic waste water through cross-flow filtration technologies		Production of chemicals, pharmaceuticals, animal feed or human food ingredients through cross-flow filtration technologies	
**Bioreactor treatment**	**Recycling of produced water**	**Bio-based formulations and materials**	**Industrial biotechnology by fermentation**
**Recycling of liquid resources**	**Treatment of oily water**	**Chemical and pharmaceutical processes**	**Filtration of sugar, starch derivatives and beverages**

What makes KLEANSEP™ unique ?

- → Ability to withstand temperatures up to 150°C (300°F)
- → High strength materials of construction
- → Compatibility with harsh chemicals (oxidizers, extreme acid or caustic, solvents, etc.)
- → Ability to withstand high concentrations of oil and solids
- → **Reliable filtration performance**
- → Tubular module: flexible membrane surface area from 0,16 m²/module to 69 m²/module
- → High durability module: housings available in a variety of materials, gaskets available in different types of elastomers

Reliable filtration performance

- Temperature up to 150°C
- Max. TransMembrane Pressure : 10 bar
- pH range : 0 – 14
- Membrane materials: oxide-based ceramic
- Housing materials: steel (316L, 316Ti, Duplex, Super-duplex, Hastelloy …), Fiber-glass (FRP) or Plastic (CPVC, …)
- Gasket materials: NBR, EPDM, FPM, SILICONE

WATER & MEMBRANES

ALSYS

This document (not contractual) and its content is the property of Orelis Environnement SAS. All rights reserved. - EN0022 - KLEANSEP CERAMIC MEMBRANES and MODULES - Data Sheet - V29/11/21

Kleansep™ membranes technical features

	Channels diameter (mm)	Membrane area (m²)	Ceramic tube length (mm)	External diameter (mm)	Number of channels	Code
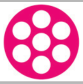	6	0,16	1178 ± 0,5 mm	25 ± 0,5 mm	7	BX
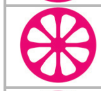	5	0,2	1178 ± 0,5 mm	25 ± 0,5 mm	8	BE
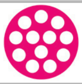	4,5	0,2	1178 ± 0,5 mm	25 ± 0,5 mm	12	BD
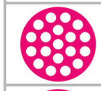	3,5	0,25	1178 ± 0,5 mm	25 ± 0,5 mm	19	BW
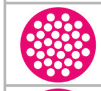	2,8	0,34	1178 ± 0,5 mm	25 ± 0,5 mm	31	BH
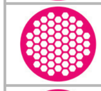	2,2	0,5	1178 ± 0,5 mm	25 ± 0,5 mm	55	BC
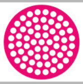	2	0,45	1178 ± 0,5 mm	25 ± 0,5 mm	61	BS

General features

Geometry:	Multichannel tubular
Ceramic support material:	Oxide-based ceramic
Ceramic membrane material:	Oxide-based ceramic
End sealing:	1 mm or 16 mm

Thermal and chemical resistance

Maximum Temperature :	Up to 150°C
Sterilization :	121°C
Sterilization by oxidizing agents :	yes

Membranes pore size and cut-off

Microfiltration:	1,0µm 0,45µm 0,2µm 0,1µm HR
Ultrafiltration:	300 kD HF, 150 kD, 50 kD, 15 kD
Nanofiltration*:	5 kD, 1 kD

*Contact us. Nanofiltration product are shaped with 19, 31 & 61 channel geometries

Mechanical and chemical resistance

Maximum TransMembrane Pressure:	10 bar
Bursting pressure:	80 bar
pH range:	0 to 14

Unaffected by solvents and radiation

Kleansep™ modules technical features

MODULES GEOMETRY		Membrane area per module depending on the membranes (m²)						
Number of membranes	Modules Code	BX	BE	BD	BW	BH	BC	BS
		7	8	12	19	31	55	61
1 membrane	Module K01	0,16	0,2	0,2	0,25	0,34	0,5	0,45
3 membranes	Module K03	0,48	0,6	0,6	0,75	1,02	1,5	1,35
7 membranes	Module K07	1,12	1,4	1,4	1,75	2,38	3,5	3,15
19 membranes	Module K19	3,04	3,8	3,8	4,75	6,46	9,5	8,55
37 membranes	Module K37	5,92	7,4	7,4	9,25	12,58	18,5	16,65
67 membranes	Module K67	10,72	13,4	13,4	16,75	24,48	33,5	30,15
99 membranes	Module K99	15,84	19,8	19,8	24,75	33,66	49,5	44,55
138 membranes	Module K138	22,08	27,6	27,6	34,5	46,92	69	62,1

Housing general features

Steel type:

- Inox 316L
- Inox 316Ti
- Titanium
- Uranus B6 (904L)
- Hastelloy C22
- Hastelloy C276

Connection type:

- Clamp
- Flange (ISO,ANSI or DIN)

Gaskets general features

Gaskets type:

- Single gasket / module
- Individual gasket / membrane

Gaskets elastomer materials:

- NBR
- EPDM
- FPM
- SILICONE

Contacts :
Europe: +33 (0)4 66 85 95 36
North America: +1 857 504 2250
Asia: +86 (0)21 6350 3377

orelis@alsys-group.com
www.alsys-group.com

This document (not contractual) and its content is the property of Orelis Environnement SAS. All rights reserved. - EN0022 - KLEANSEP CERAMIC MEMBRANES and MODULES - Data Sheet - V29/11/21
