# Supplementary figures and images for: Evaluation of Gas-to-Liquid Transfer with Ceramic Membrane Sparger for H2 and CO2 Fermentation
Source: Membranes (Basel). 2022 Dec 2;12(12):1220. doi: 10.3390/membranes12121220 (PMC9783551; doi:10.3390/membranes12121220)

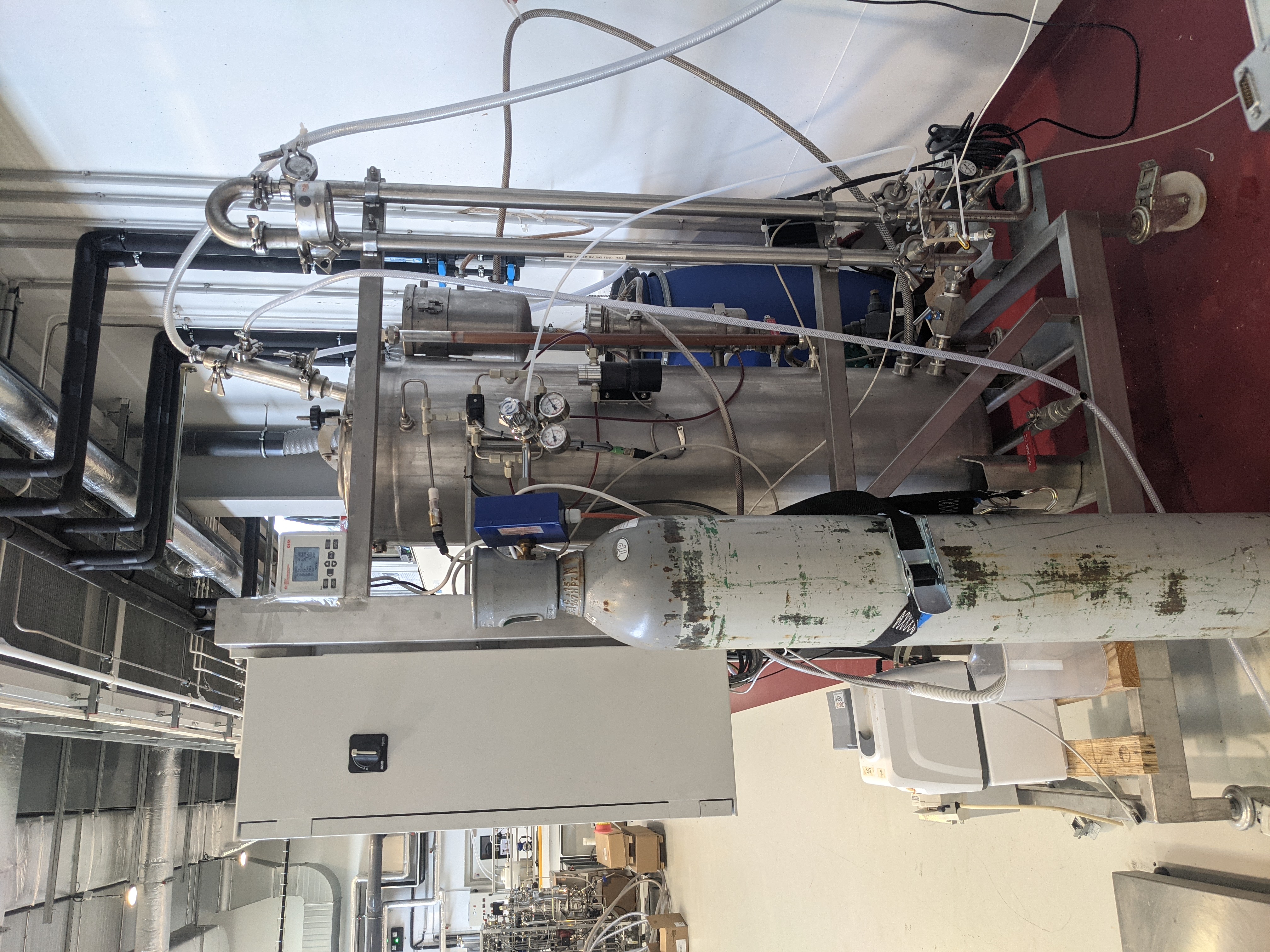

Supplement: Supplementary file 1 [file membranes-12-01220-s001.zip › Figure S2 - Pilot picture.jpg]
